# The Down-Regulation of Clusterin Expression Enhances the αSynuclein Aggregation Process

**DOI:** 10.3390/ijms21197181

**Published:** 2020-09-29

**Authors:** Chiara Lenzi, Ileana Ramazzina, Isabella Russo, Alice Filippini, Saverio Bettuzzi, Federica Rizzi

**Affiliations:** 1 Department of Medicine and Surgery, University of Parma, Via Gramsci 14, 43126 Parma, Italy; chiara.lenzi1@studenti.unipr.it (C.L.); saverio.bettuzzi@unipr.it (S.B.); federicamariaangel.rizzi@unipr.it (F.R.); 2 Centre for Molecular and Translational Oncology (COMT), University of Parma, Parco Area delle Scienze 11/a, 43124 Parma, Italy; 3 Biostructures and Biosystems National Institute (INBB), Viale Medaglie d’Oro 305, 00136 Rome, Italy; 4 Department of Molecular and Translational Medicine, University of Brescia, Via Europa 11, 25123 Brescia, Italy; isabella.russo@unibs.it (I.R.); alice.filippini@unibs.it (A.F.); 5 Genetics Unit, IRCCS Istituto Centro S. Giovanni di Dio Fatebenefratelli, Via Pilastroni 4, 25125 Brescia, Italy

**Keywords:** clusterin, αSynuclein, proteostasis, chaperone, protein aggregation, heat shock protein, neurodegeneration, gene expression

## Abstract

Parkinson’s Disease (PD) is a progressive neurodegenerative disease characterized by the presence of proteinaceous aggregates of αSynuclein (αSyn) in the dopaminergic neurons. Chaperones are key components of the proteostasis network that are able to counteract αSyn’s aggregation, as well as its toxic effects. Clusterin (CLU), a molecular chaperone, was consistently found to interfere with Aβ aggregation in Alzheimer’s Disease (AD). However, its role in PD pathogenesis has yet to be extensively investigated. In this study, we assessed the involvement of CLU in the αSyn aggregation process by using SH-SY5Y cells stably overexpressing αSyn (SH-Syn). First, we showed that αSyn overexpression caused a strong increase in CLU expression without affecting levels of Hsp27, Hsp70, and Hsp90, which are the chaperones widely recognized to counteract αSyn burden. Then, we demonstrated that αSyn aggregation, induced by proteasome inhibition, determines a strong increase of CLU in insoluble aggregates. Remarkably, we revealed that CLU down-regulation results in an increase of αSyn aggregates in SH-Syn without significantly affecting cell viability and the Unfolded Protein Response (UPR). Furthermore, we demonstrated the direct molecular interaction between CLU and αSyn via a co-immunoprecipitation (co-IP) assay. All together, these findings provide incontrovertible evidence that CLU is an important player in the response orchestrated by the cell to cope with αSyn burden.

## 1. Introduction

The cellular proteostasis network (PN) is involved in maintaining proteome integrity with the aim of ensuring cell viability under several stress conditions, including aging, exposure to environmental stress, or disease onset and progression [1]. The main components of PN are molecular chaperones. These chaperones aid in the folding and refolding of proteins, direct denatured proteins toward degradation systems, and counteract the misfolded protein aggregation process. Heat shock protein (Hsp) family members are molecular chaperones and function under both physiological and stress conditions. The failure of PN can lead to the accumulation of proteinaceous aggregates, which is a common event in different protein misfolding diseases such as Parkinson’s Disease (PD) and Alzheimer’s Disease (AD) [2,3,4].

PD is a multifactorial neurodegenerative illness characterized by the selective loss of midbrain dopaminergic neurons. The post-mortem examination of PD patients’ brains reveals the presence of intraneuronal inclusions called Lewy bodies (LB) and Lewy neurites (LN), which are both pathological hallmarks of PD. LB and LN are insoluble proteinaceous aggregates mainly composed of αSynuclein (αSyn), which is a protein that is predominantly localized at the pre-synaptic terminals of neurons. The loss of native functions of αSyn and the acquisition of toxic functions caused by the misfolding and oligomerization process plays a pivotal role in PD pathogenesis [5,6]. Increasing evidence also suggests that αSyn oligomers spread in a prion-like fashion from neuron-to-neuron, thus propagating cellular damage to nearby cells. Nevertheless, many questions about this mechanism remain unanswered [7,8].

Research strategies based on Hsp modulation are actively being explored with the aim of counteracting αSyn aggregation’s toxicity [9]. For instance, it has been shown that Hsp70, Hsp90, and other small chaperones (e.g., αβ-crystallin and Hsp27) are able to protect cells against αSyn burden [10,11]. In the present study, we investigate the role of Clusterin (CLU) on the αSyn aggregation process. CLU is a chaperone with nearly ubiquitous distribution. It is expressed in the brain, liver, and reproductive tissues [12]. CLU synthesis is governed by an orchestrated process, including epigenetic (i.e., CpG islands), transcriptional (i.e., Heat Shock Factor 1), and post-translational (i.e., N-glycosylation, proteolytic cleavage) mechanisms. As a result of the presence of a heat shock element in its promoter region, CLU is up-regulated following different stimuli [13,14,15]. CLU is known to be an extracellular ATP-independent chaperone. However, emerging data indicate that under stress conditions, intracellular CLU, in both its precursor and cleaved form, exhibits chaperone activity [16]. These CLU forms may arise from the retrotranslocation pathway triggered during cellular stress [14,16]. CLU binds client unfolded proteins, thus maintaining them in a soluble state, and presents functions similar to that of other small Hsp, such as Hsp27 and αβ-crystalline [17].

CLU is considered to be an important guardian of the brain because of its neuroprotective role in brain injury and stroke [18]. CLU is involved also in neurogenesis [19] and the neurodegenerative process [20,21,22]. At present, AD is the neuropathology in which the role of CLU has been the most extensively investigated. In vitro and in vivo studies have demonstrated that CLU binds Aβ peptides, thus preventing their aggregation and mediating their clearance through lipoprotein receptor internalization and lysosome degradation [23,24]. In addition to functional studies, Genome-Wide Association Studies (GWAS) showed that CLU is one of the major risk genes for late-onset AD [25,26]. In contrast, the role that CLU plays in PD pathogenesis has not yet been extensively elucidated. Early studies reported that CLU co-localizes with αSyn in biopsies of patients affected by α-synucleinopathies [27] and that CLU is an αSyn-associated protein in the MES cell line exposed to rotenone [28]. More recently, several independent studies found that CLU expression is up-regulated in the plasma and cerebrospinal fluid of PD patients [29,30,31]. A large PD case-control study also suggested a link between CLU and PD [32].

On the basis of the previous literature data, the focus of this research was to shed light on the possible involvement of CLU in the cellular response caused by both αSyn up-regulation and the aggregation process, prompted by MG132 treatment, in the neuroblastoma cell line. First, we characterized our SH-SY5Y cells stably overexpressing αSyn (SH-Syn) experimental models by investigating cell viability and the Unfolded Protein Response (UPR). Secondly, we studied the expression pattern of CLU compared to that of Hsp27, Hsp70, and Hsp90, which are known to take part in the PN response triggered to counteract αSyn burden. Then, we explored the possible interaction between CLU and αSyn. Lastly, we addressed the role played by CLU in the αSyn aggregation process by performing CLU short interfering RNA (siRNA) loss-of-function studies.

## 2. Results

### 2.1. Generation and Characterization of SH-Syn Cells

In the present study, we used SH-SY5Y human neuroblastoma cells to generate a cell line stably overexpressing αSyn (SH-Syn) via plasmid transfection and antibiotic selection. Control cells were also generated (SH-Mock). The rationale of our work was to study the role of CLU on the αSyn aggregation process under conditions of either mild (αSyn overexpression) or strong (αSyn overexpression and MG132 treatment) proteostasis impairment.

**Figure 1:**
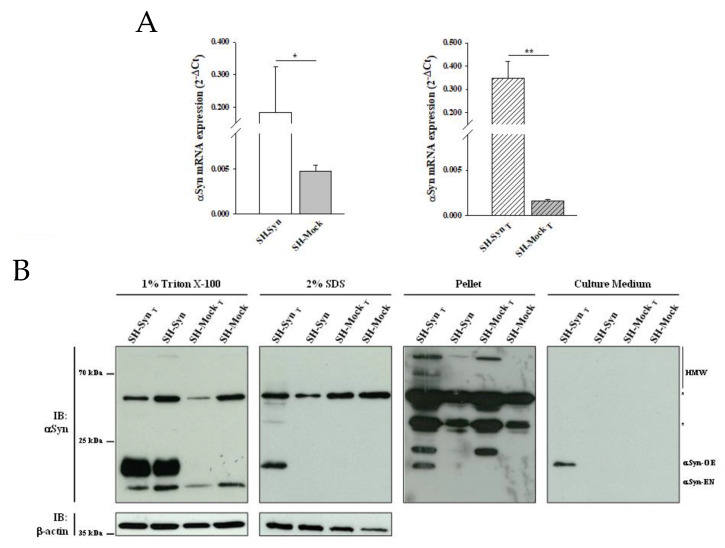
αSynuclein (αSyn) expression in SH-SY5Y cells stably overexpressing αSyn (SH-Syn) and SH-Syn_T_. (**A**) αSyn mRNA quantification by qPCR in SH-Syn and control cells (SH-Mock) (left panel) and in SH-Syn_T_ and SH-Mock_T_ (right panel). Data are presented as the means ± SD from three independent experiments, each performed in duplicate. Data were analyzed by a two-tailed Student *t*-test (* *p* < 0.05; ** *p* < 0.001). (**B**) Detection of αSyn in the 1% Triton X-100 soluble fraction, in the 2% SDS soluble fraction, in the pellet fraction, and in the cell culture medium of SH-Syn, SH-Syn_T_, SH-Mock, and SH-Mock_T_. Blots are representative of experiments repeated three times. β-actin was used as the loading control; an asterisk represents non-specific bands.

We first confirmed αSyn mRNA overexpression in SH-Syn compared to SH-Mock by qPCR analyses (Figure 1A, left panel). Then, we analyzed the presence of αSyn in different soluble and insoluble fractions using Western blot assays. According to others, monomeric αSyn is extracted in the 1% Triton X-100 fraction. Then, by increasing the detergent strength of the extraction buffer (2% SDS) to solubilize the residual pellet obtained from the first extraction, it is possible to isolate and detect the monomeric and oligomeric αSyn forms. Finally, the insoluble pellet from both the 1% Triton X-100 and 2% SDS extraction contains high molecular weight (HMW) oligomers and aggregates of αSyn [33,34,35,36]. Likewise, in the 1% Triton X-100 soluble fraction, we observed two bands of about 14 and 16 kDa, corresponding to endogenous monomeric αSyn (Syn-EN) and ectopic monomeric αSyn (Syn-OE). The increased molecular weight of the latter form is due to the presence of HA- and His-tags (Figure 1B). As expected, only the band corresponding to Syn-EN was detectable in SH-Mock. αSyn was not expressed at detectable levels in the 2% SDS soluble fraction, in the insoluble pellet, or in the cell medium in either SH-Syn or SH-Mock (Figure 1B).

SH-Syn retained a morphology similar to that of non-transfected SH-SY5Y and SH-Mock (Figure S1A). The proliferation of SH-Syn, starting from 5th and 6th day after seeding, was statistically lower than that of both SH-Mock and non-transfected SH-SY5Y (Figure S1B).

### 2.2. Effects of αSyn Overexpression on Cell Viability and UPR Induction

**Figure 2:**
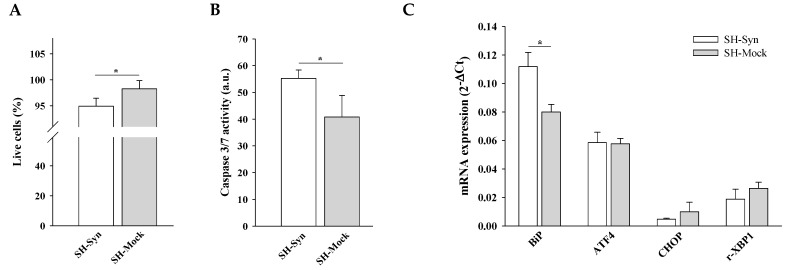
Cell viability, executioner 3/7 caspase activity, and Unfolded Protein Response (UPR) induction in SH-Syn. (**A**) Live cell count by Trypan blue staining of SH-Syn and SH-Mock. Data are presented as the means ± SD from three independent experiments, each performed in triplicate. Data were analyzed by a two-tailed Student’s *t*-test (* *p* < 0.05). (**B**) Analysis of caspase 3/7 activity in SH-Syn and SH-Mock. Data are presented as the means ± SD from two independent experiments, each performed in triplicate. Data were analyzed by a two-tailed Student’s *t*-test (* *p* < 0.05). (**C**) BiP (Binding immunoglobulin Protein), ATF4 (activating transcription factor 4), CHOP (C/EBP homologous protein), and r-XBP1 (the ratio between X-box binding protein 1′s unconventional spliced form and X-box binding protein 1′s unspliced form) mRNA quantification via qPCR in SH-Syn and SH-Mock. Data are presented as the mean ± SD of three independent experiments, each performed in duplicate. Data were analyzed by a Mann–Whitney Rank Sum Test (* *p* < 0.05).

The αSyn overexpression was correlated to a statistical reduction of viable cells (as revealed by Trypan blue cell counting, Figure 2A) and to an increase of caspase 3/7 activity (Figure 2B) in SH-Syn compared to SH-Mock.

To evaluate whether αSyn overexpression was sufficient to induce Endoplasmic Reticulum (ER) stress, we checked for the expression of UPR markers [37] by qPCR. We found that BiP (Binding immunoglobulin Protein) expression, one of the earliest sensors of UPR, was significantly increased in SH-Syn compared to SH-Mock, while ATF4 (activating transcription factor 4), CHOP (C/EBP homologous protein), and r-XBP1 (the ratio between X-box binding protein 1′s unconventional spliced form and X-box binding protein 1′s unspliced form) were not significantly increased following αSyn overexpression (Figure 2C).

### 2.3. Effects of Proteasome Inhibition on αSyn Aggregation, Cell Viability, Executioner 3/7 Caspase Activity, and UPR Induction

The imbalance of proteostasis is a crucial event in PD onset and progression. Accordingly, proteasome inhibitors are widely used to trigger αSyn aggregation [38]. Therefore, we treated SH-Syn and SH-Mock with 0.4 μM MG132, which is a concentration that corresponds to the amount of the drug required to inhibit cell viability by 50% (IC_50_) after 48 h of treatment (Figure S2). As expected, we confirmed the up-regulation of αSyn mRNA through an additional comparison between the treated SH-Syn (SH-Syn_T_) and treated SH-Mock (SH-Mock_T_) (Figure 1A, right panel).

More interestingly, the Western blot analysis revealed a different distribution of αSyn in the fractions analyzed. The presence of Syn-EN slightly decreased in the 1% Triton X-100 soluble fraction of treated cells compared to the untreated ones for both SH-Syn and SH-Mock. Notably, as a consequence of the MG132 treatment, αSyn’s monomeric, oligomeric, and HMW forms appeared in the 2% SDS and the pellet fractions of only SH-Syn_T_, indicating the formation of αSyn aggregates. Similarly, αSyn was detected exclusively in the cell culture medium of SH-Syn_T_ (Figure 1B).

**Figure 3:**
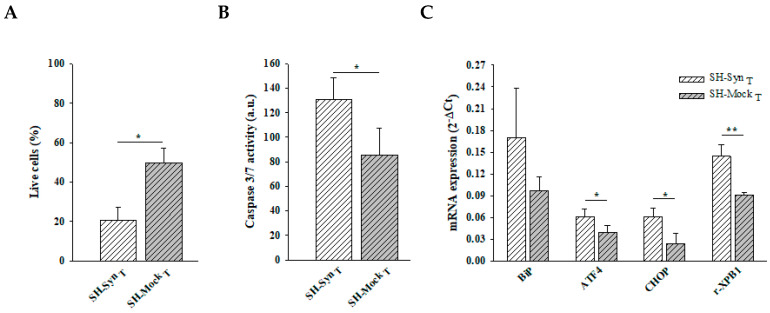
Cell viability, executioner 3/7 caspase activity, and UPR induction in SH-Syn_T_. (**A**) Live cell count by Trypan blue staining of SH-Syn_T_ and SH-Mock_T_. Data are presented as the means ± SD from three independent experiments, each performed in triplicate. Data were analyzed by a two-tailed Student’s *t*-test (* *p* < 0.05). (**B**) Analysis of caspase 3/7 activity in SH-Syn_T_ and SH-Mock_T_. Data are presented as the means ± SD from two independent experiments, each performed in triplicate. Data were analyzed by a two-tailed Student’s *t*-test (* *p* < 0.05). (**C**) BiP, ATF4, CHOP, and r-XBP1 mRNA quantification by qPCR in SH-Syn_T_ and SH-Mock_T_. Data are presented as the mean ± SD of three independent experiments, each performed in duplicate. Data were analyzed by a Mann–Whitney Rank Sum Test (* *p* < 0.05, ** *p* < 0.001).

Supporting the stressed state of the cells, Trypan blue staining revealed a statistically significant reduction of live cells in SH-Syn_T_ compared to SH-Mock_T_ (Figure 3A). Under the same experimental conditions, we found a statistically significant increase of caspase 3/7 activity (Figure 3B) concomitantly with the induction of the UPR, as revealed by the increase of ATF4, CHOP, and r-XBP1 mRNA levels in SH-Syn_T_ compared to SH-Mock_T_ (Figure 3C).

### 2.4. Expression Analyses of CLU and Other Molecular Chaperones in SH-Syn

**Figure 4:**
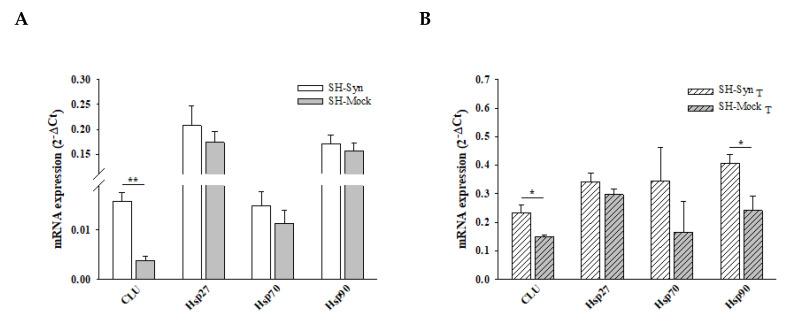
Chaperones mRNA level in SH-Syn and SH-Syn_T_. (**A**) CLU, Hsp27, Hsp70, and Hsp90 mRNA quantification by qPCR in SH-Syn and SH-Mock. (**B**) CLU, Hsp27, Hsp70, and Hsp90 mRNA quantification by qPCR in SH-Syn_T_ and SH-Mock_T_. Data are presented as the mean ± SD of three independent experiments, each performed in duplicate. Data were analyzed by a Mann–Whitney Rank Sum Test (* *p* < 0.05, ** *p* < 0.001).

**Figure 5:**
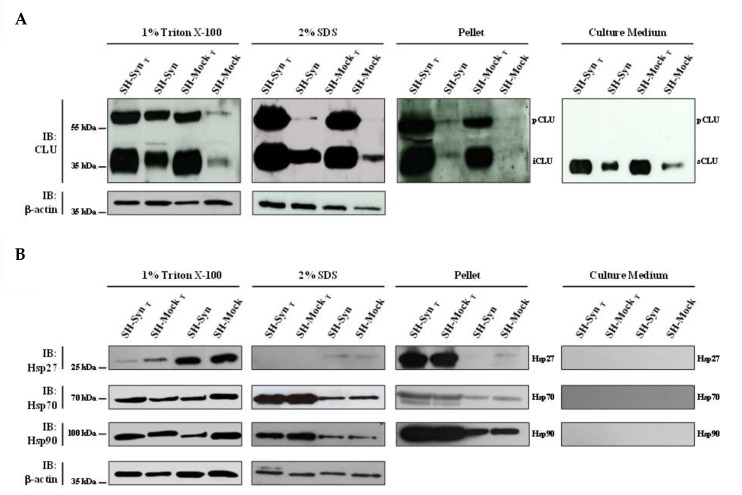
Chaperone protein level in SH-Syn and SH-Syn_T_. Detection of (**A**) CLU and (**B**) Hsp27, Hsp70, and Hsp90 in the 1% Triton X-100 soluble fraction, in the 2% SDS soluble fraction, in the pellet fraction, and in the culture media of SH-Syn, SH-Syn_T_, SH-Mock, and SH-Mock_T_. Blots are representative of experiments repeated three times. β-actin was used as the loading control.

There is an increasing body of literature that targets molecular chaperones, which are key effectors of the PN, to reduce the burden of toxic αSyn aggregates and thus slow the progression (or even stop the development) of synucleinopathies. CLU exerts its chaperone functions by inhibiting the stress-induced aggregation of different proteins and favoring misfolded protein degradation. To explore if CLU can act as a chaperone to counteract αSyn misfolding and aggregation, we investigated the expression of CLU in response to αSyn overexpression, as well as the expression following MG132 treatment. In the same experimental setting, we evaluated the expression level of other well-known chaperones that have been more extensively studied (i.e., Hsp27, Hsp70, and Hsp90). Our results clearly showed that CLU’s mRNA level was up-regulated in SH-Syn compared to SH-Mock. Interestingly, the mRNA expression of the other chaperones did not change (Figure 4A). Then, we studied the CLU protein levels by Western blot assays. As expected, in the analyzed intracellular fractions, we detected two immunoreactive bands for CLU: the precursor protein band (pCLU) of about 64 kDa and the CLU intracellular mature form of about 40 kDa (iCLU). In agreement with the qPCR results, CLU’s protein expression was higher in SH-Syn than in SH-Mock in all the analyzed fractions (Figure 5A), while the protein levels of Hsp27, Hsp70, and Hsp90 did not change (Figure 5B). Likewise, as expected, although the secreted CLU (sCLU) was clearly detectable in the cell culture medium, the other three Hsps were not detectable (Figure 5A,B).

Next, we investigated the expression profile of CLU and the other chaperones under the condition of strong proteostasis impairment due to MG132 treatment. Comparing the untreated and treated cells, the mRNA expression profile of all the chaperones significantly increased in the treated conditions (data not shown), albeit with different protein patterns among the fractions analyzed (Figure 5A,B). Notably, only the expression of CLU was increased in all the intracellular fractions and in the culture medium. Conversely, Hsp27, Hsp70, and Hsp90 levels increased only in the 2% SDS fraction and in the pellet of the MG132 treated cells compared to the untreated ones (Figure 5A,B).

Finally, we compared SH-Syn_T_ to SH-Mock_T_ to evaluate the effects of αSyn overexpression under strong proteostasis impairment conditions on the chaperones’ expression profiles_._ Strikingly, we found a significant increase in both CLU mRNA (Figure 4B) and CLU protein levels in 2% SDS and the pellet fractions, where we detected αSyn’s oligomeric and HMW forms (Figure 5A and Figure S3). In contrast, the qPCR and Western blot analyses showed that the mRNA (Figure 4B) and protein levels (Figure 5B) of Hsp27 and Hsp70 were not modified when comparing SH-Syn_T_ and SH-Mock_T_. The mRNA of Hsp90 increased in SH-Syn_T_ (Figure 4B), but its protein level did not (Figure 5B).

### 2.5. CLU and αSyn Intracellular Localization and Interaction

**Figure 6:**
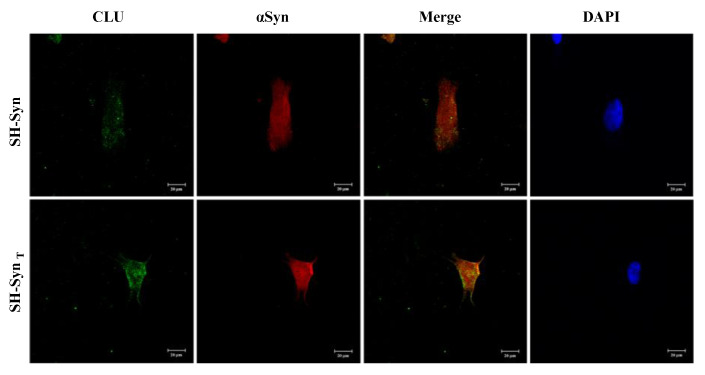
Localization of CLU and αSyn in SH-Syn and SH-Syn_T_**.** Representative images of the intracellular localization of CLU (green fluorescence) and αSyn (red fluorescence) in SH-Syn and SH-Syn_T_ acquired by confocal microscopy. Cell nuclei were stained with 4′dye-6˗diamidino˗2˗phenylindole (DAPI, blue fluorescence). Magnification 40×.

Our results strongly suggest a relationship between CLU and αSyn. Therefore, we decided to investigate by confocal microscopy analysis whether these two proteins co-localize inside the cell. In SH-Syn, the acquired images revealed a weak immunofluorescence signal with a cytoplasmic punctate pattern for CLU (green fluorescence) and a signal that was diffuse and more marked for αSyn (red fluorescence) (Figure 6). Following MG132 treatment, the cells’ morphology appeared slightly modified due to the inhibition of proteasome activity and UPR activation. Moreover, the cells exhibited a stronger signal for CLU than they did for αSyn, which is similar to the signal observed for the untreated cells as observed in the Western blot analysis. The merged signal indicated a partial co-localization of the two proteins, prevalently in the region situated in close proximity to the plasma membrane (Figure 6 and Figure S4).

**Figure 7:**
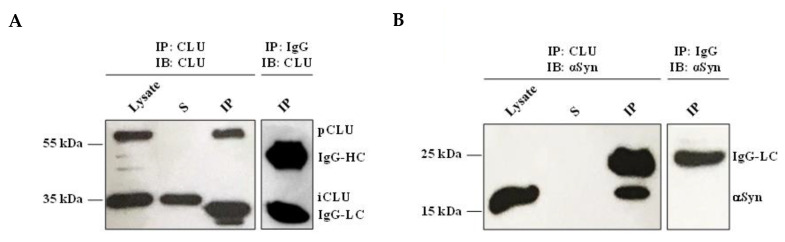
Interaction of CLU and αSyn in SH-Syn. (**A**) Detection of CLU in the SH-Syn intracellular protein fraction (Lysate) immunoprecipitated with the anti-CLU antibody. (**B**) Detection of αSyn in the SH-Syn intracellular protein fraction (Lysate) immunoprecipitated with the anti-CLU antibody. Immunoprecipitation with IgG was performed as a negative control. IP: Immunoprecipitated fraction; S: Fraction containing the proteins that do not interact with CLU; Lysate: 1% Triton X-100 protein soluble fraction of SH-Syn; IgG-HC: IgG Heavy chain; IgG-LC: IgG Light chain.

Based on the immunofluorescence analysis results, we explored the possibility of an interaction between the two proteins via a co-immunoprecipitation (co-IP) assay. We efficiently immunoprecipitated CLU from the SH-Syn cell lysate, as demonstrated by the presence of a 64 kDa band in the IP fractions (Figure 7A). The band was comparable in molecular weight and intensity to the amount of CLU present in the lysate sample that underwent co-IP. Notably, the band was absent in the surnatant fraction (S) collected after the co-IP assay (Figure 7A). An immunoreactive band, corresponding to αSyn, was detected in the IP lane when the membrane was probed with the anti-αSyn antibody, demonstrating the physical interaction between CLU and αSyn (Figure 7B). No CLU and αSyn bands were detected in the negative control sample, obtained by using the Immunoglobulin G (IgG) for the IP reaction instead of the anti-CLU antibody (Figure 7A,B).

### 2.6. Effects of CLU Down-Regulation

**Figure 8:**
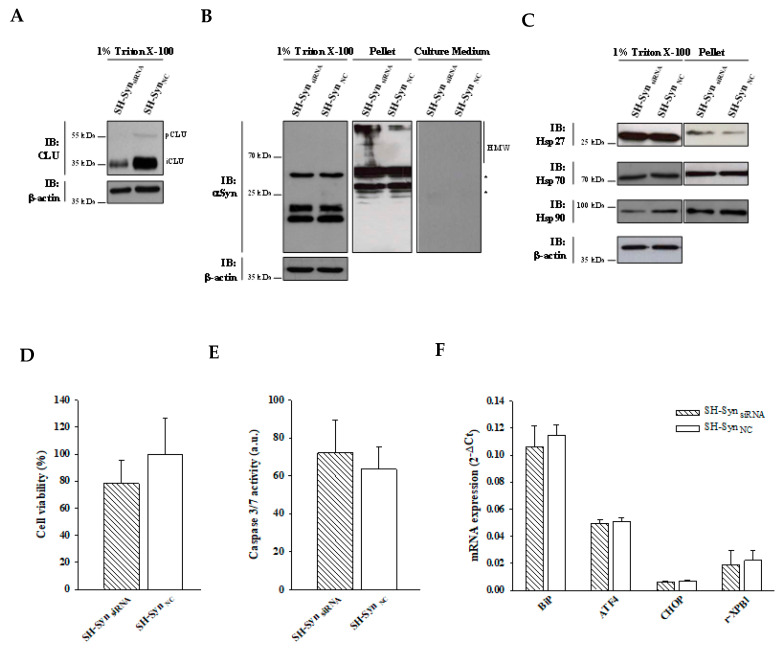
Effects of CLU down-regulation in SH-Syn. (**A**) Detection of CLU in the 1% Triton X-100 soluble fraction of SH-Syn_siRNA_ and SH-Syn_NC_. Blots are representative of experiments repeated three times. β-actin was used as the loading control. (**B**) Detection of αSyn in the 1% Triton X-100 soluble fraction, in the pellet fraction, and in the culture medium of SH-Syn_siRNA_ and SH-Syn_NC_. Blots are representative of experiments repeated two times. β-actin was used as the loading control; an asterisk represents non-specific bands. (**C**) Detection of Hsp27, Hsp70, and Hsp90 in the 1% Triton X-100 soluble fraction and in the pellet fraction of SH-Syn_siRNA_ and SH-Syn_NC_. Blots are representative of experiments repeated three times. β-actin was used as the loading control. (**D**) The cell viability of SH-Syn_siRNA_ and SH-Syn_NC_ by the WST-1 assay. Data are shown as the mean ± SD of two independent experiments, each performed in triplicate. The difference between groups is not statistically significant. (**E**) Analysis of the caspase 3/7 activity in SH-Syn_siRNA_ and SH-Syn_NC_. Data are presented as the mean ± SD of two independent experiments, each performed in triplicate. The difference between groups is not statistically significant. (**F**) BiP, ATF4, CHOP, and r-XBP1 mRNA quantification by qPCR in SH-Syn_siRNA_ and SH-Syn_NC_. Data are presented as the mean ± SD of three independent experiments, each performed in duplicate. The difference between groups is not statistically significant.

To test the hypothesis that CLU may interfere with the αSyn aggregation process, we set up loss-of-function experiments using short interfering RNA (siRNA). An effective and specific reduction of the CLU mRNA and protein levels was achieved in the siRNA transfected SH-Syn (SH-Syn_siRNA_) compared to the negative control, transfected SH-Syn (SH-Syn_NC_) (Figure 8A and Figure S5A). We found a slight increase in αSyn HMW forms in the pellet fraction of SH-Syn_siRNA_ compared to SH-Syn_NC_, while the expression of αSyn in the 1% Trition-X100 soluble fraction did not change following CLU down-regulation (Figure 8B). As expected, under this experimental condition, αSyn was not present in the cell medium at detectable levels (Figure 8B). Comparing SH-Syn_siRNA_ and SH-Syn_NC_, we also noticed that CLU down-regulation was accompanied by a slight decrease of Hsp90 in the 1% Trition-X100 soluble fraction; in contrast, the expression of Hsp27 and Hsp70 did not change (Figure 8C). Moreover, SH-Syn_siRNA_ exhibited a mild reduction (about 20%) (although not statistically significant) of cell viability compared to SH-Syn_NC_ (Figure 8D), with no significant differences in either the caspase 3/7 activity (Figure 8E) or the expression of UPR markers (Figure 8F).

**Figure 9:**
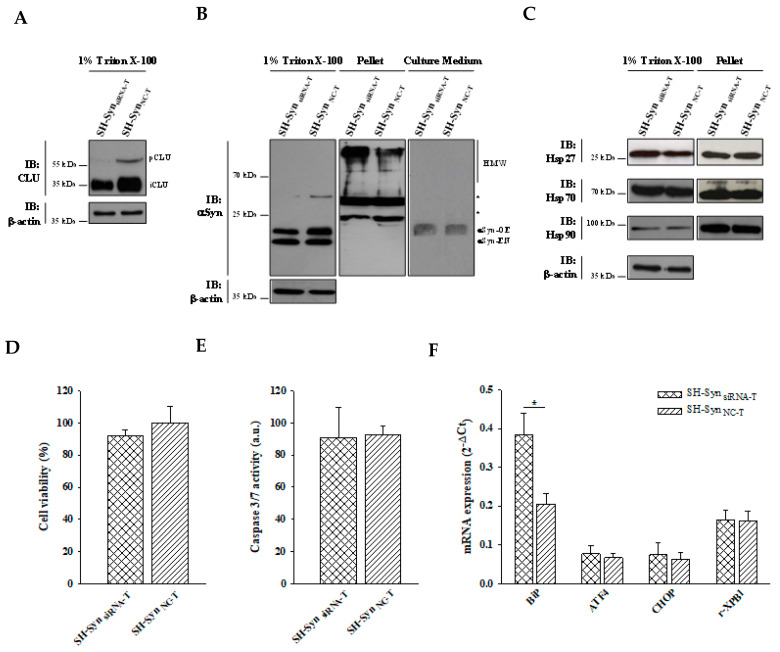
Effects of CLU down-regulation in SH-Syn_T_. (**A**) Detection of CLU in the 1% Triton X-100 soluble fraction of SH-Syn_siRNA-T_ and SH-Syn_NC-T_. Blots are representative of experiments repeated three times. β-actin was used as the loading control. (**B**) Detection of αSyn in the 1% Triton X-100 soluble fraction, in the pellet fraction, and in the culture medium of SH-Syn_siRNA-T_ and SH-Syn_NC-T_. Blots are representative of experiments repeated two times. β-actin was used as the loading control; an asterisk represents non-specific bands. (**C**) Detection of Hsp27, Hsp70, and Hsp90 in the 1% Triton X-100 soluble fraction and in the pellet fraction of SH-Syn_siRNA-T_ and SH-Syn_NC-T_. Blots are representative of experiments repeated three times. β-actin was used as the loading control. (**D**) The cell viability of SH-Syn_siRNA-T_ and SH-Syn_NC-T_ by the WST-1 assay. Data are shown as the mean ± SD of two independent experiments, each performed in triplicate. The differences between groups are not statistically significant. (**E**) Analysis of caspase 3/7 activity in SH-Syn_siRNA-T_ and SH-Syn_NC-T_. Data are presented as the mean ± SD of two independent experiments, each performed in triplicate. The difference between groups is not statistically significant. (**F**) BiP, ATF4, CHOP, and r-XBP1 mRNA quantification by qPCR in SH-Syn_siRNA-T_ and SH-Syn_NC-T_. Data are presented as the mean ± SD of two independent experiments, each performed in duplicate. Data were analyzed by a Mann–Whitney Rank Sum Test (* *p* < 0.05).

When CLU down-regulation was combined with MG132 treatment (Figure 9A and Figure S5B), we observed a different distribution of αSyn in the analyzed fractioning buffers. In particular, αSyn’s HMW form levels were markedly increased in the pellet, while the monomeric αSyn form slightly decreased in the 1% Triton X-100 soluble fraction of the treated SH-Syn_siRNA_ (Syn_siRNA-T_) compared to the treated SH-Syn_NC_ (SH-_SynNC-T_). As expected, αSyn was also detected in the extracellular environment but without a significant difference between SH-Syn_siRNA-T_ and SH-Syn_NC-T_ (Figure 9B). Under these conditions, we found that CLU down-regulation was not accompanied by significant changes of the other chaperones investigated in this study (Figure 9C). Lastly, we observed a significant increase of Bip expression in SH-Syn_siRNA-T_ compared to SH-Syn_NC-T_, without a significant increase of the other effectors involved in the UPR (Figure 9F). Likewise, cell viability (Figure 9D) and caspase 3/7 activities (Figure 9E) were not significantly affected by CLU down-regulation.

## 3. Discussion

Over the past years, many advances have been made in understanding the molecular mechanisms leading to the onset and progression of PD. However, new targets for novel therapies aimed at limiting the disease’s progression are urgently needed. One of the most promising approaches to counteracting the αSyn aggregation process and subsequent toxicity is modulation of the expression of molecular chaperones. Chaperones are relevant PN players because they are potentially able to prevent, and eventually even reverse, protein misfolding and aggregation.

In the present study, we investigated the role played by CLU in an αSyn overexpressing cell line model that mimics the conditions of both mild and strong proteostasis impairment induced by MG132 treatment. We established, for the first time, that CLU is part of the biochemical mechanism triggered by the cell to manage αSyn overexpression, which is a condition yielding mild proteostasis impairment. More specifically, we clearly showed in SH-Syn that CLU expression is up-regulated earlier and more strongly than Hsp27, Hsp70, and Hsp90—the chaperones most extensively studied in neurodegenerative diseases [10,11]. This observation is biologically relevant because it suggests that CLU transcription and translation is evoked by the cells when the αSyn level is increased. Consistent with other studies, we found that αSyn overexpression was not a sufficient stimulus to induce αSyn aggregation but sufficed to up-regulate the UPR regulator Bip [39,40], to reduce cell viability and to activate apoptosis through a caspases dependent mechanism [41,42,43,44].

We further studied the role played by CLU on αSyn aggregation by treating cells with MG132 to induce strong proteostasis impairment [38]. As expected, we observed the presence of αSyn HMW forms in the 2% SDS soluble buffer and in the pellet of SH-Syn_T_, confirming that the αSyn aggregation process occurred. A large increase in ER stress was also evident, as revealed by the increased expression of all the UPR markers (PERK and IRE1α UPR-branches) and the increase in the activity of caspase 3/7, which is in agreement with other studies performed on different experimental models [43,45]. Comparing SH-Syn_T_ and SH-Mock_T_, we demonstrated an increase of the CLU level in the same fraction in which we detected the αSyn HMW forms, while the expression of Hsp27, Hsp70, and Hsp90 did not change. In summary, although all the analyzed chaperones were up-regulated by MG132 treatment, only CLU up-regulation was specifically driven by αSyn aggregation, showing that this phenomenon was not a simple bystander effect of proteasome inhibition. Furthermore, the co-presence of CLU and αSyn in the culture media of SH-Syn_T_ suggests that this chaperone may also be involved in the uptake of αSyn species from the extracellular space. Notably, this mechanism is also likely to take place in PD, similar to what has been proposed for the clearance of the Aβ peptide in AD [23,46,47]. The possibility that CLU might act in the extracellular milieu is particularly relevant, considering that many authors have proposed that αSyn oligomers spread in a prion-like fashion from cell to cell, thereby expanding the neurotoxic damage to proximal regions [7,8]. However, the intracellular fate of the misfolded protein uptake mediated by CLU is not yet completely elucidated. In support of CLU’s involvement in the αSyn aggregation process, we found that CLU and αSyn co-localized inside the cell and that αSyn is a CLU-client protein, as revealed by their physical interactions in the IP assay. Although the interactions between these two proteins was already observed in a cell-free model [48,49], to the best of our knowledge, this is the first time that this binding has been confirmed in a cell model mimicking the conditions of αSyn burden.

Although several studies explored, using overexpression experiments, the ability of different chaperones to cope with αSyn burden, very few studies evaluated their ability to modulate the αSyn aggregation pathway throughout loss-of-function investigations [50,51]. To prove the functional involvement of CLU in the αSyn aggregation process, we down-regulated CLU expression via siRNA. We detected an increase of αSyn HMW forms when CLU expression was down-regulated under conditions of only αSyn overexpression and also when the proteome’s instability was enhanced. Under the latter conditions, we observed an increase in Bip, supporting the idea that a decrease of CLU expression promotes the αSyn aggregation process and aggravates ER stress. Interestingly, CLU down-regulation did not cause a modification of Hsp27 or Hsp70 expression, suggesting the lack of any compensatory effects between the Hsp. In contrast, CLU down-regulation led to a decreased expression of Hsp90. This event may underline a cause-and-effect relationship between these two chaperones. However, further studies are necessary to clarify their possible interplay. In our experimental model, CLU down-regulation and the resulting increase of αSyn aggregates did not have significant effects on either cell viability or caspase activity. However, we cannot rule out the possibility that these results are due to the point in time in which we performed the analyses, as a longer treatment period could uncover more significant cytotoxic effects. 

Overall, we demonstrated for the first time that CLU is part of the biochemical response triggered by the cell to manage αSyn overexpression and that CLU down-regulation favors or exacerbates the αSyn aggregation process, suggesting that this chaperone is involved in the dynamic αSyn aggregation process. Additional studies are needed to articulate the molecular mechanisms that explain CLU’s ability to counteract the αSyn aggregation process and to explore CLU as a potential therapeutic target for PD onset and progression.

## 4. Materials and Methods

### 4.1. Cell Culture and Transfection

Human neuroblastoma SH-SY5Y cells were purchased from ATCC (Manassas, VA, USA) and maintained at 37 °C in a humidified atmosphere supplied with 5% CO_2_ in a Dulbecco’s Modified Eagle Medium (DMEM):Ham’s F12 medium (1:1) (Gibco^®^, Life Technologies, Carlsbad, Canada) supplemented with 10% Fetal Bovine Serum (FBS), 2 mM L-glutamine, and 100 U/mL penicillin and 100 g/mL streptomycin (Lonza, Basel, Switzerland). Cell harvesting was performed using a Trypsin/Ethylenediaminetetraacetic acid (EDTA) solution (Sigma-Aldrich, Steinheim, Germany).

The cells were seeded in 60 mm dishes (1.5 × 10^6^ cells/dish) and transfected with pHM6-αSyn-wt (#40824, AddGene, Cambridge, MA) or pHM6-Mock using a K2^®^ Transfection System (Biontex Laboratories GmbH, München, Germany) to generate SH-SY5Y stably overexpressing αSyn (SH-Syn) or control cells (SH-Mock), respectively. G418 at 800 μg/mL (Sigma-Aldrich, Steinheim, Germany) was added to the culture medium 48 h after transfection for transfected cell selection; then, the cells were maintained in a culture medium supplemented with G418 at 200 μg/mL.

Cell morphology phase-contrast images were acquired via an inverted microscope Zeiss Axiovert 200 (Carl Zeiss, Gottingen, Germany) equipped with a color digital camera Axiocam MR (Carl Zeiss, Gottingen, Germany) using the Axiovision 4.8 program (Vysis, Downers Grove, IL, USA).

### 4.2. Cell Viability Assay

The cells were seeded in a 96-well plate (30 × 10^3^ cells/well) and allowed to attach overnight. Thereafter, the cells were treated with increasing concentrations of MG132 (0.2–2.0 µM). After 48 h of treatment, cell viability was determined with the WST-1 assay (Figure S6) in accordance with the manufacturer’s protocol (Roche, Lewes, UK). Dose–response curves were generated, and the IC_50_ value was determined by a non-linear regression analysis (a four-parameter logistic curve) using the SigmaPlot software (version 12.0). The WST-1 assay was also used to determine cell viability after the siRNA experiments (Figure S6).

### 4.3. Trypan Blue Staining

The cells were seeded in 35 mm dishes (750 × 10^3^ cells/dish), and after 72 h, 25 μL of Trypan blue was added to 25 μL of the cell suspension; then, 10 μL of the obtained mixture was transferred to the Burker chamber to enable the counting of vital cells (non-stained cells) and non-vital cells (stained cells). Trypan blue staining was also performed after 0.4 µM MG132 treatment for 48 h (Figure S6).

### 4.4. Cell Proliferation Assay

The cell proliferation was evaluated by a crystal violet assay. The cells were seeded in a 6-well plate (35 × 10^3^ cells/well), and an assay was performed 48, 72, 96, 120, and 144 h after seeding. At the end of each established time point, the cells were washed with 1× Phosphate-buffered saline (PBS) and fixed with 4% paraformaldehyde in 1× PBS for 20 min at room temperature. Then, the cells were incubated with a solution containing 0.5% crystal violet (Sigma-Aldrich, Steinheim, Germany) in 20% methanol for 15 min. After careful washing with H_2_O, the dye was extracted with a 0.1 M sodium citrate solution in 50% ethanol (pH 4.2) and quantified at 540 nm with an EnSpire^®^ Multimode Plate Reader instrument (PerkinElmer, Waltham, MA, USA).

### 4.5. siRNA Experiments

CLU silencing was carried out by transfecting cells with an siRNA sequence (5′˗GCAGCAGAGUCUUCAUCAU˗3′, Ambion, Austin, TX, USA) complementary to a portion of the exon 2 of the CLU sequence and was able to simultaneously silence all its transcriptional variants (siRNA-CLU). The control condition was represented by cells transfected with a siRNA scrambled sequence (Integrated DNA Technologies, Coralville, CA, USA) that did not bind to any mRNAs inside the cell (siRNA-NC). The cells were seeded in 35 mm dishes (750 × 10^3^ cells/dish) and transfected with 100 nM of siRNA-CLU or siRNA-NC using a Trans-IT-TKO Transfection Reagent (Mirus Bio, Madison, WI, USA) in accordance with the manufacturers’ instructions. Cells were harvested 24 h after transfection and used for further analyses (Figure S6). In the CLU down-regulation experiment combined with MG132 treatment, after transfection, the cells were incubated with 0.4 μM MG132 for 24 h (Figure S6).

### 4.6. Caspases Assay

A Caspase-Glo^®^ 3/7 assay kit (Promega, Madison, WI, USA) was used to evaluate the activity of caspases 3/7. Briefly, the cells were seeded in a 96-well plate (30 × 10^3^ cells/well). After 72 h, the activity of caspase 3/7 was determined in accordance with the manufacturer’s protocol. In the MG132 treatment and siRNA experiments, the caspase 3/7 activity was determined at an established time point (Figure S6). The luminescent signal was measured by the EnSpire^®^ Multimode Plate Reader instrument (PerkinElmer, Waltham, MA, USA) and normalized for DNA content, determined by a CellTox^TM^ Green Cytotoxicity Assay kit (Promega, Madison, WI, USA). The fluorescent signal was measured with EnSpire^®^ Multimode Plate Reader instruments (PerkinElmer, Waltham, MA, USA).

### 4.7. RNA Extraction and qPCR

The cells were seeded in 60 mm dishes (1.5 × 10^6^ cells/dish). After 72 h, the cells were washed with 1X PBS and lysed with 1 mL TRIzol Reagent (Fisher Molecular Biology, Rome, Italy). In the MG132 treatment and siRNA experiments, the cells were lysed at an established time point (Figure S6). The RNA extraction and purification were performed using a PureLink^®^ RNA Mini Kit (Ambion, Austin, TX, USA) in accordance with the manufacturer’s protocol. The RNA obtained was quantified by a spectrophotometer (Eppendorf, Hamburg, Germany) and checked for quality and integrity via electrophoretic analyses. RevertAid Reverse Transcriptase was used to obtain the cDNA (Thermo Fisher Scientific, Waltham, MA, USA). For each reaction, at 500 ng of RNA, 0.2 μg of random primers was added in a final volume of 12.5 µL. The obtained mixture was incubated for 5 min at 65 °C. Subsequently, 4 µL of Reaction Buffer 5×, 1 mM Deoxynucleotide triphosphates mix (dNTPs) (Sigma-Aldrich, Steinheim, Germany), and 1 µL of RevertAid Reverse Transcriptase were added in a final volume of 20 µL. Then, each reaction was incubated for 10 min at 25 °C, 60 min at 45 °C, and 10 min at 70 °C. The generated cDNA was diluted 1:2 and amplified by qPCR using specific primers (Table S1). The enzyme glyceraldehyde 3-phosphate dehydrogenase (GAPDH) was chosen as the reference gene. The amplification reactions were performed using 2 µL of cDNA, 10 µL of SsoAdvanced^TM^ Universal SYBR^®^ Green Supermix (Bio-Rad, Berkley, CA, USA), and 0.2 µM of Forward and Reverse primers in a final reaction volume of 20 µL. Amplifications were performed with an MJ Opticon 4 Instrument (MJ Research, Waltham, MA, USA). For each analysis, the samples were analyzed in duplicate. The quantity of each mRNA was calculated via the 2^˗∆Ct^ method, where ∆Ct was obtained by subtracting the Ct of the housekeeper gene from the threshold cycle (Ct) of the target gene.

### 4.8. Sequential Extraction and Western Blot Analyses

To separate the soluble and insoluble αSyn fractions, the method described in [34] was used with minor modifications. Briefly, the cells were seeded in 60 mm dishes (1.5 × 10^6^ cells/dish). After 72 h, the cells were washed with 1X PBS and collected using a Radioimmunoprecipitation assay buffer (RIPA buffer, 50 mM Tris-HCl pH 7.4, 100 mM NaCl, 1% Triton X-100) supplemented with cocktails of protease and phosphatase inhibitors (Sigma-Aldrich, Steinheim, Germany). In the MG132 treatment and siRNA experiments, the cells were harvested at an established time point (Figure S6). The cell lysate was stirred at 4 °C for 1 h and then centrifuged at 12,000 rpm at 4 °C for 30 min to recover the supernatant containing the soluble proteins in the 1% Triton X-100 soluble fraction. The pellet was resuspended in RIPA supplemented with 2% SDS, vortexed at room temperature for 30 min, and centrifuged at 12,000 rpm at 4 °C for 45 min to recover the supernatant containing the soluble proteins in a 2% SDS buffer (2% SDS soluble fraction). The obtained pellet (insoluble fraction) was resuspended in the loading buffer for the SDS˗PAGE analyses. The concentration of soluble proteins was determined by a DC Protein assay (Bio-Rad, Hercules, CA, USA) using bovine serum albumin (Sigma-Aldrich, Steinheim, Germany) as a standard. The absorbance was measured at 750 nm using an EnSpire^®^ Multimode Plate Reader instrument (PerkinElmer, Waltham, MA, USA).

For the Western blot analysis, 50 µg of intracellular proteins or 30 µL of the culture medium were resolved on 10% or 14% polyacrylamide gel (SDS˗PAGE) and then transferred on a 0.45 µm Polyvinylidene fluoride (PVDF) membrane (Millipore, Billerica, MA, USA). The transfer efficiency was verified by 0.1% Red Ponceau S (Sigma-Aldrich, Steinheim, Germany) staining. The membranes were incubated at room temperature for 3 h with 5% non-fat dry milk (Sigma-Aldrich, Steinheim, Germany) in Tris-buffered saline with 0.1% Tween^®^ 20 detergent (TTBS) to block the non-specific binding sites and probed overnight at 4 °C with a primary antibody by gentle shaking (Table S2). Then, the membranes were incubated with suitable secondary antibodies (Table S2) conjugated to horseradish peroxidase for 1 h at room temperature. Immunoreactive bands were detected using a Luminata^TM^ Crescendo Western HRP Substrate (Millipore, Billerica, MA, USA).

### 4.9. Double-Immunofluorescence Analysis

Cells were seeded on a coverslip in a 24-well plate (160 × 10^3^ cells/well). Seventy-two hours after seeding or after 48 h of MG132 treatment (Figure S6), the cells were washed in D-PBS (Lonza, Basel, Switzerland), fixed with 4% paraformaldehyde in 1× PBS for 12 min, and permeabilized with 0.1% Triton X-100 in Tris-buffered saline (TBS) 1× at room temperature for 12 min. The slides were transferred to a humidified chamber, and non-specific binding sites were blocked with 5% Bovine serum albumin (BSA) (Sigma-Aldrich, Steinheim, Germany) in D-PBS at room temperature for 45 min. Subsequently, the cells were probed with the anti-CLU primary antibody (Table S2) for 1 h at room temperature. After two washings, labeling was performed with the Alexa Fluor 488-conjugated secondary antibody (Invitrogen, Carlsbad, CA, USA) at room temperature for 1 h while shielded from ambient light. Then, the blocking phase was repeated as previously reported, followed firstly by incubation with the anti-αSyn primary antibody (Table S2) and then by the Alexa Fluor 568-conjugated secondary antibody (Invitrogen; Carlsbad, CA, USA). The nuclei were stained with DAPI (Sigma-Aldrich, Steinheim, Germany) and embedded in Mowiol (Sigma-Aldrich, Steinheim, Germany). Fluorescence images were acquired with a laser confocal microscope system (LSM 510 Meta scan head integrated with an Axiovert 200 M inverted microscope; Carl Zeiss, Jena, Germany).

### 4.10. Co-Immunoprecipitation Assay

In total, 1 mg of proteins in 1 mL of RIPA buffer supplemented with protease and phosphatase inhibitors was pre-cleared with 20 μL of beads of Protein G PLUS-Agarose (Santa Cruz Biotechnology, Dallas, TX, USA), keeping the sample in orbital rotation at 4 °C for 30 min. Then, the lysate was separated from the beads by centrifugation at 2500 rpm at 4 °C for 5 min, and 30 μL was stored as the input sample. The pre-cleared lysate was placed in orbital rotation with 25 μg of anti-CLU primary antibody (Table S2) at 4 °C for 1 h followed by incubation with 20 μL of beads at 4 °C for 16 h. After centrifugation at 2500 rpm at 4 °C for 5 min, the supernatant (S) containing the proteins that did not interact with the protein of interest were collected in a new tube. The immunoprecipitated fraction (IP) was washed four times with 1 mL RIPA, resuspended in a Laemmli buffer, incubated at 100 °C for 5 min to perform a reverse cross-link reaction, and, finally, centrifuged at 2500 rpm at room temperature. As a negative control, an immunoprecipitation reaction was also performed using IgG. The samples were subjected to Western blot analysis according to the protocol described previously.

### 4.11. Statistical Analysis

Data are expressed as the mean values ± SD for the indicated number of independent determinations. All statistical analyses were performed using the SigmaPlot software (Version 12.0). A two-tailed Student *t*-test or Mann–Whitney Rank Sum Test was used to compare the two groups, and a one-way ANOVA test followed by a Holm–Sidak multiple comparison post-hoc test was used to determine the differences between more than two groups; *p* values < 0.05 were considered statistically significant.

## Supplementary Materials

The following are available online at https://www.mdpi.com/1422-0067/21/19/7181/s1. Figure S1. Morphological and cell proliferation analyses. Figure S2. MG132 cytotoxicity analysis. Figure S3. Densitometric analysis of CLU protein levels in SH-Syn_T_. Figure S4. Localization of CLU and αSyn in SH-Syn_T_. Figure S5. CLU down-regulation in SH-Syn and SH-Syn_T_. Figure S6. The experiments time lines. Table S1. Sequences of the primers used in qPCR analysis. Table S2. List of antibodies used.

## Abbreviations

αSyn	αSynuclein
αSyn-EN	Endogenous Syn
αSyn-OE	Overexpressed Syn
Aβ	Amyloid-beta peptide
AD	Alzheimer’s Disease
ATF4	Activating transcription factor 4
BiP	Binding Immunoglobulin Protein
CHOP	C/EBP homologous protein
CLU	Clusterin
co-IP	Co-immunoprecipitation
DAPI	4′dye-6˗diamidino˗2˗phenylindole
GAPDH	Glyceraldehyde 3˗phosphate dehydrogenase
GWAS	Genome-Wide Association Study
HMW	High molecular weight
HSF-1	Heat shock factor-1
IB	Immunoblotted
iCLU	Intracellular CLU
IP	Immunoprecipitated fraction
IRE1α	Inositol-requiring enzyme 1 alpha
LB	Lewy bodies
LN	Lewy neuritis
MES	Mesencephalic/neuroblastoma hybrid cell line
pCLU	CLU precursor protein
PD	Parkinson’s Disease
PERK	PKR-like ER kinase
qPCR	Quantitative real-time PCR
r-XBP1	XBP1us/XBP1
sCLU	CLU secreted protein
SH-SY5Y	Human neuroblastoma cell line
SH-Mock	SH-SY5Y cells transfected with pHM6-mock
SH-Mock_T_	MG132 treated control cells
SH-Syn	SH-SY5Y cells transfected with pHM6-αSyn-wt
SH-Syn_NC_	NC-transfected SH-Syn
SH-Syn_NC-T_	MG132 treated NC-transfected SH-Syn
SH-Syn_siRNA_	CLU siRNA-transfected SH-Syn
SH-Syn_siRNA-T_	MG132 treated CLU siRNA-transfected SH-Syn
SH-Syn_T_	MG132 treated SH-Syn
UPR	Unfolded Protein Response
XBP1	X-box binding protein 1
XBP1us	X-box binding protein 1 unconventional spliced form
