# Supplementary material for: The Down-Regulation of Clusterin Expression Enhances the αSynuclein Aggregation Process
Source: Int J Mol Sci. 2020 Sep 29;21(19):7181. doi: 10.3390/ijms21197181 (PMC7582711; doi:10.3390/ijms21197181)
Supplement: Supplementary file 1 [file ijms-21-07181-s001.pdf]

The Down-regulation of Clusterin Expression Enhances the αSynuclein Aggregation Process

Chiara Lenzi, Ileana Ramazzina*, Isabella Russo, Alice Filippini, Saverio Bettuzzi &
Federica Rizzi

List of the material included:

Supplementary Figure 1. Morphological and cell proliferation analyses.

Supplementary Figure 2. MG132 cytotoxicity analysis.

Supplementary Figure 3. Densitometric analysis of CLU protein levels in SH-Syn$_T$.

Supplementary Figure 4. Localization of CLU and αSyn in SH-Syn$_T$.

Supplementary Figure 5. CLU down-regulation in SH-Syn and SH-Syn$_T$.

Supplementary Figure 6. The experiments timelines.

Table S1. Sequences of the primers used in qPCR analysis.

Table S2. List of antibodies used.

References.

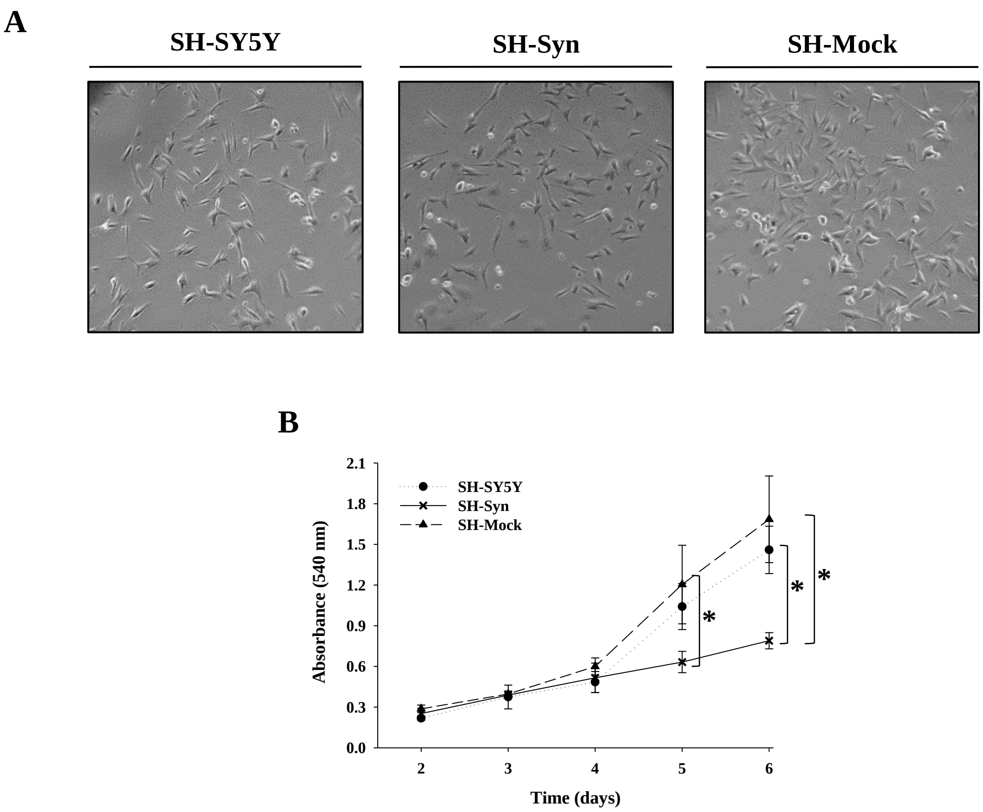

Supplementary Figure 1. Morphological and cell proliferation analyses. (A) SH-SY5Y (left panel), SH-Syn (middle panel) and SH-Mock (right panel) cells morphology in phase contrast microscopy. **(B)** Proliferation rates of SH-SY5Y, SH-Syn and SH-Mock by crystal violet assay. Data are presented as the mean ± SD from three independent experiments, each performed in triplicate. Data were analyzed by a One-way ANOVA test followed by a Holm-Sidak multiple comparison test to compare cell lines (*$p < 0.05$).

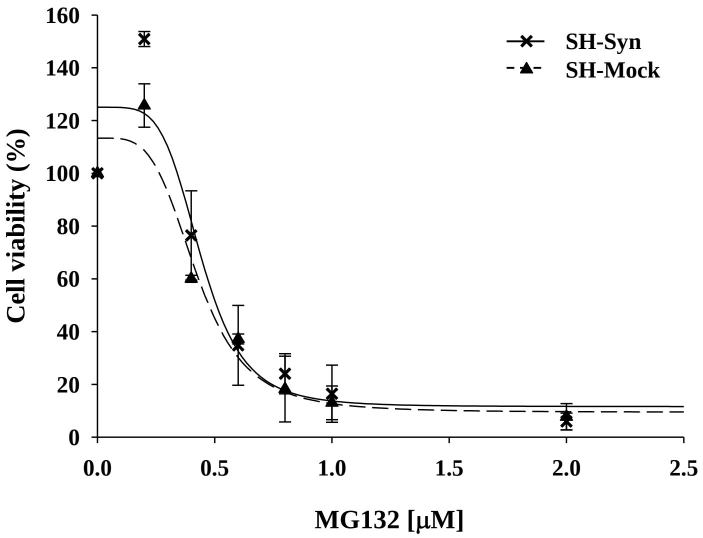

Supplementary Figure 2. MG132 cytotoxicity analysis. SH-Syn and SH-Mock cell viability analyzed by WST-1 assay after 48 hours of MG132 treatment. Dose-response curves were generated and IC_{50} were determined by a non-linear regression analysis (four parameter logistic curve). Data are presented as the mean ± SD from three independent experiments, each performed in triplicate.

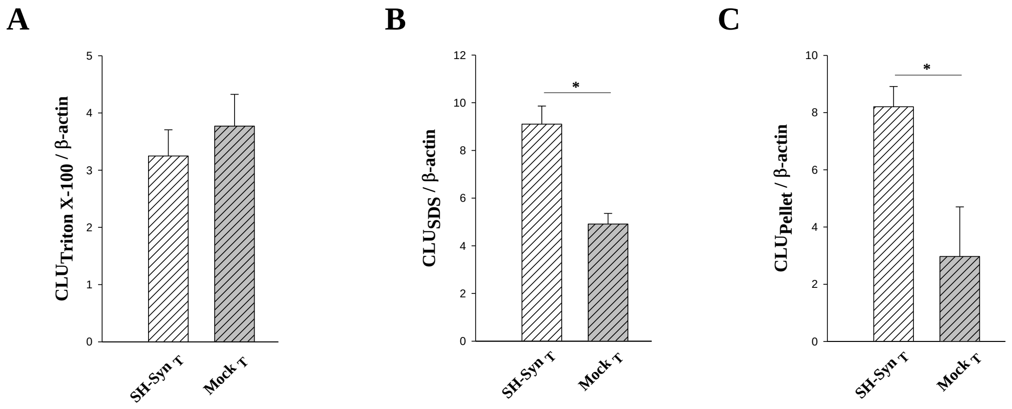

Supplementary Figure 3. Densitometric analysis of CLU protein levels in SH-Syn$_T$. CLU levels normalized to β-actin in the **(A)** 1% Triton X-100 soluble fraction, **(B)** 2% SDS soluble fraction and **(C)** pellet. Data are presented as the mean ± SD from three independent experiments (*$p < 0.05$).

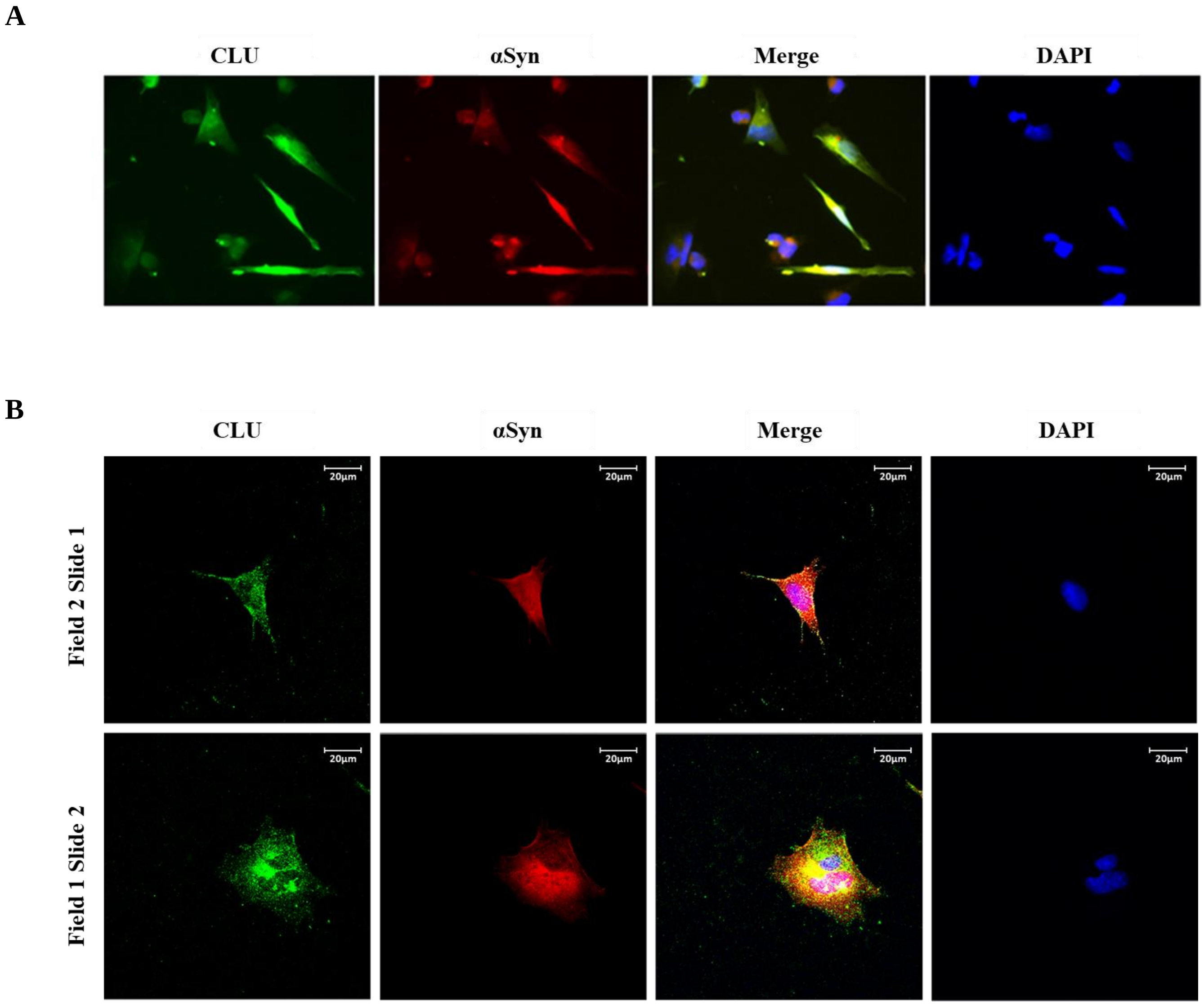

Supplementary Figure 4. Localization of CLU and αSyn in SH-Syn$_T$. Representative images of intracellular localization of CLU (green fluorescence) and αSyn (red fluorescence) in SH-Syn$_T$ acquired by **(A)** normal fluorescence microscopy (Magnification 20X) and **(B)** confocal microscopy (Magnification 40X). Cell nuclei were stained with DAPI (blue fluorescence).

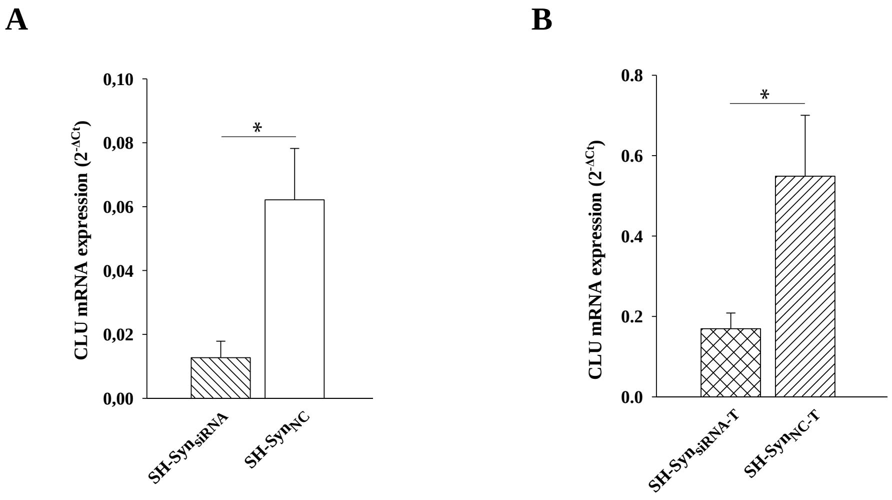

Supplementary Figure 5. CLU down-regulation in SH-Syn and SH-Syn$_T$. (A) CLU mRNA quantification in SH-Syn$_{siRNA}$ and SH-Syn$_{NC}$. Data are presented as the mean ± SD from three independent experiments, each performed in duplicate. Data were analyzed by a Mann-Whitney Rank Sum Test (*$p < 0.05$). **(B)** CLU mRNA quantification in SH-Syn$_{siRNA\text{-}T}$ and SH-Syn$_{NC\text{-}T}$. Data are presented as the mean ± SD from three independent experiments, each performed in duplicate. Data were analyzed by a Mann-Whitney Rank Sum Test (*$p < 0.05$).

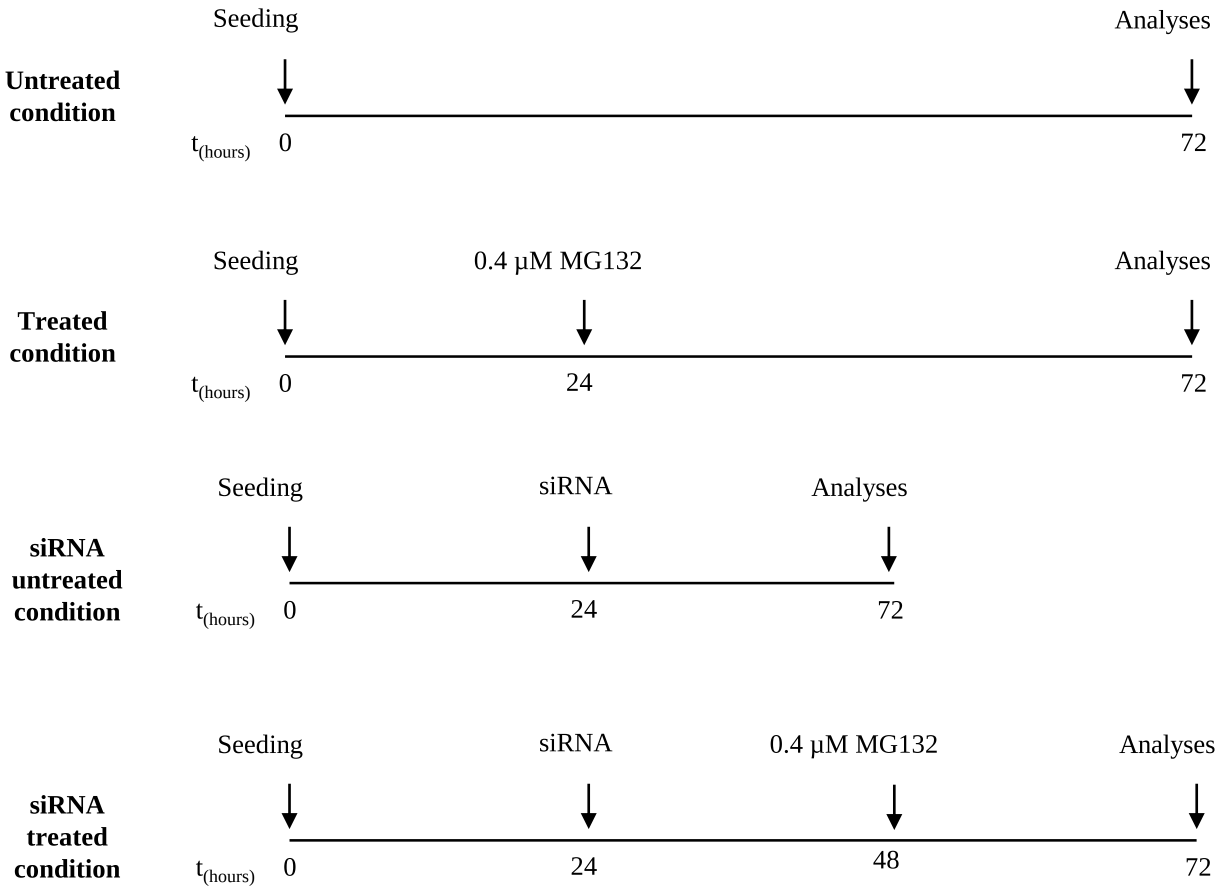

Supplementary Figure 6. The experiments timelines. Details of the experimental conditions and timelines of the analyses reported in the article.

Table S1. Sequences of the primers used in qPCR analysis.

mRNA	Primer Forward 5’→3’	Primer Reverse 5’→3’	T °C annealing	Cycles
αSyn [1]	CAACAGTGGCTGAGAAGACCA	CTCCTTCTTCATTCTTGCCCA	60	40
CLU [2]	TGATCCCATCACTGTGACGG	GCTTTTTGCGGTATTCCTGC	60	40
Hsp27 [3]	AAGTTTCCTCCTCCCTGTCC	CGGGCTAAGGCTTTACTTGG	60	40
Hsp70 [3]	GGAGGCGGAGAAGTACA	GCTGATGATGGGGTTACA	60	40
Hsp90 [3]	GATAAACCCTGACCATTCC	AAGACAGGAGCGCAGTTTCATAAA	60	40
Bip [4]	GCCGTCCTATGTCGCCTTC	TTTGTTTGCCCACCTCCAAT	58	40
ATF4 [5]	ATGACCGAAATGAGCTTCCTG	CTGGAGAACCCATGAGGTTTG	58	40
CHOP [4]	CTTCTCTGGCTTGGCTGACT	TCCCTTGGTCTTCCTCCTCT	58	40
XBP1-total [4]	CCTTGTAGTTGAGAACCAGG	GGAAGGGCATTTGAAGAACA	58	40
XBP1-us [4]	GCTGAGTCCGGCAGGTGC	GGAAGGGCATTTGAAGAACA	58	40
GAPDH [2]	AACCTGCCAAATATGATGAC	TTGAAGTCAGAGGAGACCAC	60	40

Table S2. List of antibodies used.

	Antibody	Species	Technique and dilution
Primary	Anti-αSyn (Clone 42, BD Transdunction Laboratories)	Mouse	WB: 1:500 in Milk 5%<br>IF: 1:50 in BSA 3%
	Anti-CLUα (SC-6420, Santa Cruz Biotechnology)	Goat	WB: 1:1.000 in Milk 5%<br>IF: 1:10 in BSA 3%
	Anti-CLU Human (AF2937, R&D System)	Goat	IP: 25 µg/mL
	Anti-Hsp27 (SC-13132, Santa Cruz Biotechnology)	Mouse	WB: 1:500 in Milk 5%
	Anti-Hsp70 (ab181606, Abcam)	Rabbit	WB: 1:2.000 in Milk 5%
	Anti-Hsp90 (ADI-SPA-830, Enzo Life Sciences)	Mouse	WB: 1:500 in Milk 5%
	Anti-βactin (SC:81178, Santa Cruz Biotechnology)	Mouse	WB: 1:500 in Milk 5%
Secondary	Anti-Mouse IgG (A5906, Sigma-Aldrich)	Sheep	WB: 1:5.000 in Milk 5%
	Anti-Goat IgG (A8919, Sigma-Aldrich)	Rabbit	WB: 1:5.000 in Milk 5%
	Anti-Rabbit IgG (A0545, Sigma-Aldrich)	Goat	WB: 1:200.000 in Milk 5%
	Anti-Goat IgG (Alexa Flour™ 488, Invitrogen)	Rabbit	IF: 1:300 in BSA 3%
	Anti-Mouse IgG (Alexa Flour™ 568, Invitrogen)	Goat	IF: 1:300 in BSA 3%

WB: western blot assay; IF: immunofluorescence assay; IP: immunoprecipitation assay.

REFERENCES

1. Asi, Y.T.; Simpson, J.E.; Heath, P.R.; Wharton, S.B.; Lees, A.J.; Revesz, T.; Houlden, H.; Holton, J.L. Alpha-synuclein mRNA expression in oligodendrocytes in MSA. *Glia* **2014**, *62*, 964–970, doi:10.1002/glia.22653.

2. Bonacini, M.; Coletta, M.; Ramazzina, I.; Naponelli, V.; Modernelli, A.; Davalli, P.; Bettuzzi, S.; Rizzi, F. Distinct promoters, subjected to epigenetic regulation, drive the expression of two clusterin mRNAs in prostate cancer cells. *Biochim. Biophys. Acta - Gene Regul. Mech.* **2015**, *1849*, doi:10.1016/j.bbagrm.2014.11.003.

3. Kilpatrick, K.; Novoa, J.A.; Hancock, T.; Guerriero, C.J.; Wipf, P.; Brodsky, J.L.; Segatori, L. Chemical induction of Hsp70 reduces α-synuclein aggregation in neuroglioma cells. *ACS Chem. Biol.* **2013**, *8*, 1460–1468, doi:10.1021/cb400017h.

4. Ishimura, S.; Furuhashi, M.; Mita, T.; Fuseya, T.; Watanabe, Y.; Hoshina, K.; Kokubu, N.; Inoue, K.; Yoshida, H.; Miura, T. Reduction of endoplasmic reticulum stress inhibits neointima formation after vascular injury. *Sci. Rep.* **2014**, *4*, 4–11, doi:10.1038/srep06943.

5. Tardito, S.; Isella, C.; Medico, E.; Marchiò, L.; Bevilacqua, E.; Hatzoglou, M.; Bussolati, O.; Franchi-Gazzola, R. The thioxotriazole copper (II) complex A0 induces endoplasmic reticulum stress and paraptotic death in human cancer cells. *J. Biol. Chem.* **2009**, *284*, 24306–24319, doi:10.1074/jbc.M109.026583.
